# Definitive lobectomy without frozen section analysis is a treatment option for large or deep nodules selected carefully with clinical diagnosis of malignancy

**DOI:** 10.1111/1759-7714.13493

**Published:** 2020-05-22

**Authors:** Shohei Mori, Yuki Noda, Takamasa Shibazaki, Daiki Kato, Hideki Matsudaira, Jun Hirano, Takashi Ohtsuka

**Affiliations:** ^1^ Division of Thoracic Surgery, Department of Surgery The Jikei University School of Medicine Tokyo Japan

**Keywords:** Frozen sections, lung neoplasms, pulmonary surgical procedures, sensitivity and specificity

## Abstract

**Background:**

Tissue harvesting for patients with a lung nodule is sometimes unsuitable due to the size and location of the nodule. In such cases, it is unclear whether it is acceptable to proceed to definitive lobectomy without intraoperative frozen section analysis.

**Methods:**

We retrospectively reviewed patients who underwent definitive lobectomy or wedge resection for frozen section analysis at our institution between 2014 and 2018. The sensitivity, specificity, and accuracies of the clinical and frozen section diagnoses were evaluated against the final pathological diagnosis.

**Results:**

There were 141 patients in the definitive lobectomy group and 58 patients in the frozen section analysis group, with the latter having smaller and less deep nodules and a lower rate of malignancy on clinical and final pathological diagnoses. The sensitivity, specificity, and accuracy of the clinical diagnosis were 100%, 82%, and 95%, respectively, in the frozen section analysis group and 99%, 67%, and 97%, respectively, in the definitive lobectomy group; values of frozen section diagnosis were 98%, 82%, and 93%, respectively. On subgroup analysis, all ground‐glass nodules clinically diagnosed as malignant had a final pathological diagnosis of malignancy.

**Conclusions:**

The accuracy of the clinical diagnosis was high and was not inferior to the frozen section diagnosis. These data suggest that definitive lobectomy is an acceptable treatment option for carefully selected patients with large or deep nodules and ground‐glass nodules clinically diagnosed as malignant. To avoid unnecessary lobectomy, frozen section diagnosis should be considered for nodules likely to be benign.

**Key points:**

**Significant findings of the study**

The accuracy of the clinical diagnosis was high and was not inferior to the frozen section diagnosis.

**What this study adds**

Definitive lobectomy is an acceptable treatment option for carefully selected patients with large or deep nodules and ground‐glass nodules with a clinical diagnosis of malignancy.

## Introduction

Computed tomography (CT) is effective for screening patients at a high risk of lung cancer, increasing the likelihood of identifying a solitary pulmonary nodule.^1^, ^2^, ^3^ After assessment of the probability of malignancy, when clinical pretest probability and radiological findings are discordant or the probability of malignancy is low to moderate, a non‐surgical biopsy such as a transbronchial biopsy or a transthoracic needle biopsy is considered to proceed to definitive management.^4^, ^5^, ^6^, ^7^ However, when in relation to the size and location of the nodule, tissue harvesting may be unsuitable. A surgical biopsy is considered if false‐negative results are still suspected after a non‐surgical biopsy or performing a non‐surgical biopsy would expose the patient to unnecessary risks or inadequately lengthen surgical management.^4^, ^5^

For patients with a lung nodule without a preoperative tissue diagnosis, radical surgery, generally lobectomy, is often performed after a diagnosis of malignancy from intraoperative frozen section analysis (FSA), with the tissue specimen obtained by wedge resection or needle biopsy.^8^, ^9^ Nevertheless, a considerable problem remains because of the 2%–13% false‐negative rate using FSA^9^, ^10^ and the potential risk for tumor dissemination in cases with a positive margin after wedge resection or needle biopsy.^11^, ^12^ A few studies have reported initial definitive radical surgery in the absence of tissue diagnosis as an option for selected patients, reducing cost and shortening operative time and length of hospital stay.^13^, ^14^, ^15^ However, it remains unclear whether it is acceptable to proceed to definitive lobectomy (DL) without intraoperative FSA.

Therefore, the aim of our study was to clarify whether DL could be an acceptable treatment option in selected cases by evaluation of diagnostic accuracy of clinical and frozen section diagnosis among patients who underwent DL or wedge resection for FSA.

## Methods

### Patients and data collection

We retrospectively reviewed the medical records of consecutive patients who underwent lung resection without a preoperative tissue diagnosis between January 2014 and December 2018 at the Jikei University Hospital in Tokyo, Japan. Of these, patients who underwent DL or wedge resection for FSA with intended follow‐up lobectomy were potential cases for our study, and we excluded patients with coexisting or a past‐history of other malignancy, including lung malignancy (with the intention of eliminating the possibility of metastatic lung tumors), and those who underwent pneumonectomy, bilobectomy, segmentectomy, wedge resection without intraoperative FSA, and surgery without curative intent (with the intention to evaluate definitive resection without FSA in lobectomy performed most frequently as a radical resection).

**Figure 1:**
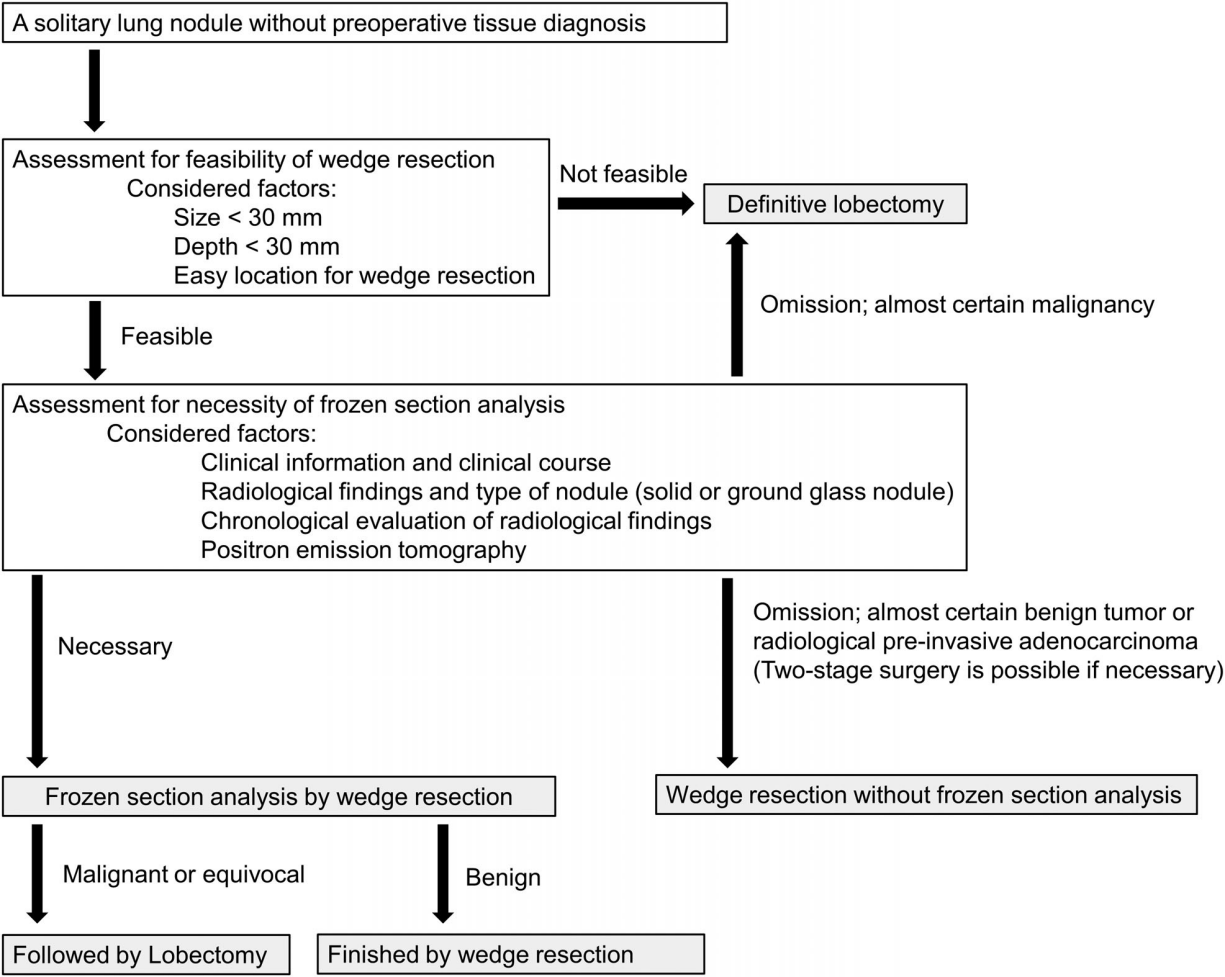
Surgical treatment algorithm for lung nodules without preoperative tissue diagnosis.

The clinical diagnosis was based on a comprehensive review of the clinical information, clinical course, radiological findings (spiculation, pleural indentation, shape of the nodule, ground‐glass nodule [GGN], and calcification pattern), chronological evaluation of radiological findings at multiple times including tumor doubling time, and fluorodeoxyglucose‐positron emission tomography (FDG‐PET). Patients were selected based on the degree of certainty of ensuring a safe margin for wedge resection and on the probability of malignancy. They were subsequently assigned to the group of candidates either for DL or for wedge resection for FSA according to our surgical treatment algorithm for lung nodules without preoperative tissue diagnosis (Fig 1).

Clinical demographic and perioperative information and pathological diagnosis were included in the analysis using the following variables collected from the medical records and our prospective database: age, sex, smoking history, radiological findings of lung CT and FDG‐PET, any preoperative biopsy, preoperative clinical diagnosis, clinical stage, the result of frozen section diagnosis, number of stapler cartridges used during the wedge resection, margins of the wedge resection, operative time for wedge resection and FSA, surgical approach and procedure, final pathological diagnosis and stage, total operative time, and postoperative complications. From CT findings, types of nodules were classified as pure GGN, part‐solid GGN, or solid nodule. The malignant probability score, which is applicable to cases with solid nodules, reported by Herder *et al*. was calculated by variables of age, smoking, history of extrathoracic cancer, tumor diameter, existence of spiculation, tumor located in the upper lobe, and the results of FDG‐PET.^16^ This scoring method incorporating FDG‐PET as a variable was reported to be more reliable than other scoring methods. Clinical and pathological staging were classified according to the eighth edition of the TNM Classification of Malignant Tumors.

Informed consent was obtained from all patients for treatment, after explaining the surgical indication for DL and wedge resection for FSA with intended follow‐up lobectomy, the associated risks and benefits. All procedures were performed in accordance with the ethical standards of the Ethics Committee of Jikei University School of Medicine (date: 13 May 2019, approval number: 31‐015[9514]), and the Declaration of Helsinki and its later amendments. The need for obtaining individual patient consent was waived due to the retrospective design of the study.

### Statistical analysis

We compared demographics, perioperative outcomes (including operative time, rate of postoperative complications, and length of postoperative hospital stay), and pathological diagnoses between the DL and FSA groups using Fisher's exact test or Mann‐Whitney U test as appropriate for the data type and distribution. Perioperative outcomes of wedge resection without lobectomy in cases of benign frozen section diagnosis were excluded from the analysis to eliminate the effects of the extent of surgical resection. Sensitivity, specificity, accuracy, positive predictive, and negative predictive values were calculated for clinical diagnosis or frozen section diagnosis against the final pathological diagnosis. On a subgroup analysis, these values were also compared between the GGN, including pure and part‐solid and solid nodule groups.

All analyses were performed using JMP (version 13.0.0; SAS Institute, Cary, NC, USA), and a *P*‐value < 0.05 was considered statistically significant.

## Results

**Figure 2:**
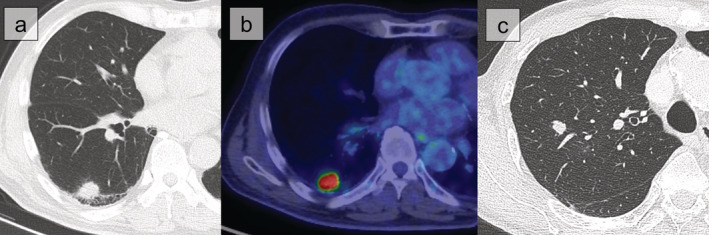
Computed tomography (CT) scan and fluorodeoxyglucose‐positron emission tomography of an example case with high malignant probability score in the definitive lobectomy group (**a**, **b**) and CT scan of an example case for which preoperative non‐surgical biopsy was unsuitable in the frozen section diagnosis group (**c**).

In total, 818 patients underwent lung resection between January 2014 and December 2018 at our institute. Of these, 199 patients without preoperative tissue diagnosis met our selection criteria and were included in our analysis: 141 in the DL group and 58 in the FSA group. Table 1 shows individual reasons of the patients assigned to the DL group according to our surgical treatment algorithm. Fig 2a,b shows the CT and FDG‐PET scan results of an example case with high malignant probability score in the DL group and Fig 2c shows the CT scan results of an example case for which preoperative nonsurgical biopsy was unsuitable in the FSA group.

**Table 1:** Individual reasons of 141 patients assigned to definitive lobectomy according to our surgical treatment algorithm for lung nodules without preoperative tissue diagnosis

Individual reason	*n* (%)
Assessment for feasibility of wedge resection	
Not feasible due to nodule size	6 (4)
Not feasible due to nodule depth	64 (45)
Not feasible due to both of size and depth of nodule	28 (20)
Assessment for necessity of frozen section analysis	
Omission because of almost certain malignancy	43 (31)

Seven cases that exceeded the size or depth criteria were not assigned to definitive lobectomy because of the easy location of the nodule to enable wedge resection.

Patient characteristics, perioperative information, and pathological diagnoses for each group are shown in Table 2. Patients in the FSA group had smaller and less deep nodules, a higher rate of solid nodules, a higher rate of no uptake and lower rate of intense uptake on FDG‐PET, a lower malignant probability score, and a lower rate of malignancy on clinical and final pathological diagnosis than those in the DL group. The comparison of perioperative outcomes for patients treated by lobectomy indicated a longer operative time for the FSA than the DL group. In the FSA group, a median of three stapler cartridges was used (standard deviation 1), with a median wedge resection time of 37 minutes (standard deviation 22) and a median time for FSA (from sample submission to reporting of results) of 32 minutes (standard deviation 19). A positive surgical margin, on paraffin section analysis, was identified in five cases, with four of these cases being malignant.

**Table 2:** Patients' background for the two groups: the definitive lobectomy group and wedge resection for the frozen section analysis with intended follow‐up lobectomy group

			Frozen section analysis group (*n* = 58)		Definitive lobectomy group (*n* = 141)		
			*n*	(%)	*n*	(%)	*P*‐value
Age (years), Median ± SD			67 ± 12		68 ± 10		0.821
Sex	Male		32	(55)	85	(60)	0.529
	Female		26	(45)	56	(40)	
Smoking history	Yes		37	(64)	95	(67)	0.625
	No		21	(36)	46	(33)	
Radiological findings	Type of nodule	Solid nodule	39	(67)	78	(55)	0.042
		Part‐solid GGN	19	(33)	56	(40)	
		Pure GGN	0	(0)	7	(5)	
	Spiculation	Yes	6	(10)	20	(14)	0.644
		No	52	(90)	121	(86)	
	Diameter, mm (Median ± SD)		16 ± 8		24 ± 11		< 0.001
	Depth, mm (Median ± SD)		20 ± 9		34 ± 15		< 0.001
	Location	Upper lobe	26	(45)	77	(55)	0.217
		Elsewhere	32	(55)	64	(45)	
PET	Intense uptake		4	(7)	31	(22)	0.007
	Moderate uptake		12	(20)	26	(18)	
	Faint uptake		16	(28)	44	(31)	
	No uptake		8	(14)	4	(3)	
	No PET study		18	(31)	36	(26)	
Malignant probability score	>65%		15	(38)	59	(76)	< 0.001
	5–65%		16	(41)	17	(22)	
	<5%		8	(21)	2	(2)	
Preoperative non‐surgical biopsy	Yes		5	(9)	28	(20)	0.060
	No		53	(91)	113	(80)	
Preoperative clinical diagnosis	Malignancy		44	(76)	134	(95)	< 0.001
	Stage 0		2	(3)	7	(5)	
	Stage 1		41	(71)	107	(76)	
	Stage 2		1	(2)	14	(10)	
	Stage 3		0	(0)	6	(4)	
	Benign		14	(24)	7	(5)	
Frozen section diagnosis	Malignant		41	(70)			
	Equivocal		2	(4)			
	Benign		15	(26)			
Surgical approach	Thoracoscopic surgery		54	(93)	127	(90)	0.723
	Convert to thoracotomy		1	(2)	5	(4)	
	Thoracotomy		3	(5)	9	(6)	
Procedure	Right upper lobectomy		17	(29)	54	(38)	< 0.001
	Right middle lobectomy		3	(5)	10	(7)	
	Right lower lobectomy		12	(20)	26	(19)	
	Left upper lobectomy		3	(5)	23	(16)	
	Left lower lobectomy		8	(14)	28	(20)	
	Wedge resection		15	(26)	0	(0)	
Final pathological diagnosis	Malignancy		41	(71)	132	(94)	< 0.001
	Adenocarcinoma		30	(52)	101	(72)	< 0.001
	Squamous cell carcinoma		8	(14)	19	(13)	
	Neuroendocrine carcinoma		2	(3)	8	(6)	
	Other		1	(2)	4	(3)	
	Benign		17	(29)	9	(6)	
Operative time (minutes), Median, SD			265 ± 52		228 ± 63		0.002
Postoperative complication	Yes		7	(16)	31	(22)	0.521
	No		36	(84)	110	(78)	
Postoperative hospital stay (days), Median, SD			6 ± 2		7 ± 15		0.090

Malignant probability was calculated in cases of solid nodule.

Operative time, postoperative complication, and length of hospital stay were analyzed in cases of lobectomy.

GGN, ground‐glass nodule; PET, positron emission tomography; SD, standard deviation.

**Figure 3:**
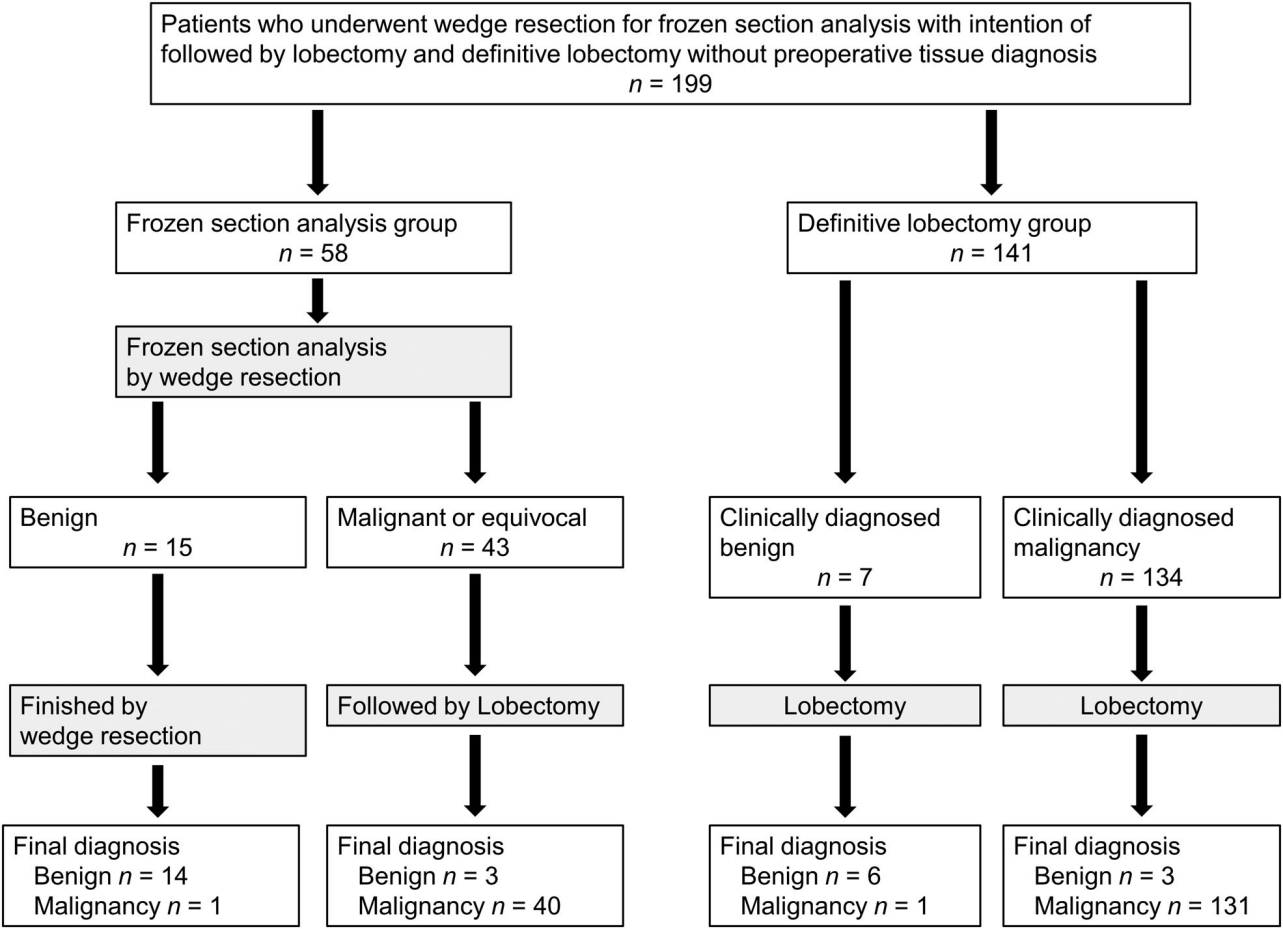
Flowchart showing the frozen section diagnosis in the frozen section analysis group and clinical diagnosis in the definitive lobectomy group, as well as the final pathological diagnosis in each group.

The flowchart of frozen section diagnosis, clinical diagnosis, and the final pathological diagnoses in each group is shown in Fig 3. In the FSA group, 15 cases had a benign diagnosis on FSA by wedge resection and did not undergo lobectomy. Of these, the final pathological diagnosis was adenocarcinoma in one case and benign in the remaining 14 cases. The 43 patients with a malignant or equivocal diagnosis (41 and two cases, respectively) on FSA by wedge resection were treated with curative lobectomy. Of these, 3 were benign and the remaining 40 cases were malignant on final pathological diagnosis.

In the DL group, seven cases with a benign clinical diagnosis, for whom wedge resection was unsuitable due to the size or depth of the nodule, were treated by lobectomy to confirm pathologically a definitive benign diagnosis. Their final pathological diagnoses were adenocarcinoma in one case and benign in the remaining six cases. The 134 cases in the DL group with a malignant clinical diagnosis were treated with lobectomy, and three of these cases were benign tumor and the remaining 131 cases were malignant on final pathological assessment. There were 43 cases that were feasible for wedge resection but were omitted and assigned to the DL group because of almost certain malignancy according to our algorithm described above, and they all received a final pathological diagnoses of malignancy.

Overall, an error in clinical diagnosis was identified in three cases in the FSA group and four in the DL group, while there were four cases with an error in the FSA by wedge resection. The details of these diagnostic errors are presented in Table 3.

**Table 3:** Details of errors in clinical and frozen section diagnosis

Error type	Sex	Age (years)	Diameter (mm)	Depth (mm)	Uptake of PET	Malignant probability (%)	Clinical diagnosis	Frozen section diagnosis	Final pathological diagnosis
Definitive lobectomy group									
Clinical diagnosis	Male	53	34	64	Moderate	96	Malignant		Organizing pneumonia
Clinical diagnosis	Male	70	35	52	Moderate	94	Malignant		Inflammatory pseudotumor
Clinical diagnosis	Female	59	11	45	No uptake	2	Malignant		Localized inflammatory lung disease
Clinical diagnosis	Male	40	13	50	Faint	10	Benign		Adenocarcinoma
Frozen section analysis group									
Clinical and frozen section diagnosis	Male	78	18	10	Intense	78	Malignant	Equivocal	Interstitial pneumonia
Frozen section diagnosis	Male	70	27	17	Faint	39	Benign	Malignant	Necrotic nodules with mycobacterium infection
Frozen section diagnosis	Male	65	63	63	No PET study	99	Benign	Equivocal	Unusual mesenchymal alveolar tumor
Frozen section diagnosis	Male	79	13	22	No PET study	38	Malignant	Benign	Adenocarcinoma
Clinical diagnosis	Male	48	22	27	Moderate	77	Malignant	Benign	Granulomatous inflammation
Clinical diagnosis	Female	76	14	20	No uptake	2	Malignant	Benign	Fibrotic scar

Error cases were all solid nodule.

PET, positron emission tomography.

Diagnostic accuracies of clinical and frozen section diagnosis are shown in Table 4. Both had equivalently high diagnostic accuracy. On subgroup analysis by nodule type (Table 5), all GGN (pure and part‐solid) cases that were diagnosed clinically malignant were classified as malignant on final pathological diagnoses; the accuracy was 100%. The accuracy of frozen section diagnosis of GGNs was also 100%.

**Table 4:** Diagnostic accuracies of clinical and frozen section diagnosis

	Sensitivity (%)	Specificity (%)	Accuracy (%)	Positive predictive value (%)	Negative predictive value (%)
Clinical diagnosis					
All (*n* = 199)	99	77	96	97	95
Definitive lobectomy group (*n* = 141)	99	67	97	98	86
Frozen section analysis group (*n* = 58)	100	82	95	93	100
Frozen section diagnosis (*n* = 58)	98	82	93	93	93

**Table 5:** Diagnostic accuracies of clinical and frozen section diagnosis in each subgroup of nodule type

	Sensitivity (%)	Specificity (%)	Accuracy (%)	Positive predictive value (%)	Negative predictive value (%)
Pure GGN and part‐solid GGN					
Clinical diagnosis (*n* = 82)	100	N/A	100	100	N/A
Frozen section diagnosis (*n* = 19)	100	N/A	100	100	N/A
Solid nodule					
Clinical diagnosis (*n* = 117)	99	77	94	94	95
Frozen section diagnosis (*n* = 39)	95	82	90	88	93

GGN, ground‐glass nodule.

## Discussion

There were two major findings in this study. The diagnostic accuracy of clinical diagnosis was high and was not inferior to that of frozen section diagnosis, and the accuracies of frozen section and clinical diagnosis for GGN were both 100%.

First, the diagnostic accuracy of clinical diagnosis was high and not inferior to that of frozen section diagnosis in both groups. The FSA group included smaller and less deep nodules and a higher rate of benign clinical diagnosis than the DL group. Based on these factors, FSA by wedge resection was deemed to be feasible and necessary for these patients. Diagnostic accuracy, sensitivity, and specificity between the clinical and frozen section diagnoses were not different in the FSA group. The benign nature of the nodule in the 14 cases with a benign clinical diagnosis was diagnosed by FSA and confirmed by final pathological diagnosis, avoiding unnecessary lobectomy. One patient with a benign frozen section diagnosis had a malignant final pathological diagnosis, and the error was caused by sampling, whereas three cases with a malignant or equivocal frozen section diagnosis had a benign pathological diagnosis, and the error was caused by interpretation. Previous studies reported false‐negative and false‐positive rates of FSA against the paraffin section (final) diagnosis ranging between 1.9%–13.1% and 0%–0.2%, respectively, with a specifically higher false‐negative rate for small nodules (≤1.1 cm in diameter).^9^, ^10^ Other studies also reported on the errors and pitfalls of FSA.^8^, ^17^, ^18^ Surgeons should be aware of the possibility of these diagnostic errors, even with FSA. The DL group included nodules likely to be almost malignant or larger and deeper nodules that were unsuitable for wedge resection for FSA. For these cases, the accuracy of the clinical diagnosis was high and not inferior to that of frozen section diagnosis and it would be acceptable and reasonable to perform DL without FSA. Previous studies also supported performing DL without preoperative tissue diagnosis and FSA for patients with a high probability of malignant nodules, and this study added information about the accuracy of clinical diagnosis.^13^, ^14^, ^15^ The specificity of the clinical diagnosis was inferior to that of FSA in our study, probably due to the small number of benign nodules on the final diagnosis in the DL group. A small number of lobectomies for nodules with clinical benign diagnosis that were unsuitable for wedge resection due to their size or depth were performed to confirm benign on a final pathological diagnosis.

Second, the diagnostic accuracy of frozen section and clinical diagnosis for GGNs (pure and part‐solid) were 100%. Therefore, GGNs selected carefully with a clinical malignant diagnosis would be enough reason to proceed to definitive resection such as lobectomy or segmentectomy without the need for confirmation of malignancy by FSA. Diagnostic accuracy of FSA by wedge resection was also 100%. However, because of concerns of the possibility of a positive margin in cases of wedge resection for large or deep GGNs with extensive and unclear boundaries, which carry the risk of dissemination,^12^ these cases should be candidates for lobectomy.

Intraoperative core needle biopsy for FSA provides an alternative method to wedge resection for large or deep nodules, which are unsuitable for wedge resection. Isaka *et al*.^19^ reported a sensitivity, specificity, and accuracy of 94%, 88%, and 94%, respectively, for a core needle biopsy. However, because FSA using intraoperative core needle biopsy has the possibility for not only diagnostic error but also sampling error due to small and deep lesions, as well as a potential risk of pleural dissemination,^11^ this procedure was not performed as standard. We noted that four malignant cases (7%) in the wedge resection for FSA had a positive surgical margin. Therefore, the potential risk for pleural dissemination is not negligible and the indications for FSA by wedge resection should be carefully considered including taking the size and depth of the nodule and radiological findings predicting malignancy into consideration.

The limitations of our study should be acknowledged. First, we used a retrospective design, comparing groups with very different backgrounds. The DL group included large and high malignant probability nodules or many GGNs, which would affect the high diagnostic accuracy. Furthermore, because FSA was not performed in all patients, the diagnostic accuracy of frozen section diagnosis was among the patients assigned to the FSA group which included small and low malignant probability nodules or less GGNs. Second, we did not include cases of segmentectomy as an oncologically acceptable resection. However, because oncologically acceptable segmentectomy is generally indicated for GGNs,^20^, ^21^, ^22^, ^23^ our results may support oncologically acceptable segmentectomy without intraoperative FSA for GGNs. Third, we did not include wedge resection for GGNs to assess the invasiveness of adenocarcinoma on paraffin section analysis (for final pathological diagnosis) to determine the need for secondary lobectomy later. A recent study reported on the evaluation of invasiveness of adenocarcinoma using FSA.^24^ If preinvasive adenocarcinoma can be evaluated with high accuracy, the clinical significance of FSA may be beyond benign/malignant judgment.

In conclusion, the accuracies of clinical diagnosis in both groups were high and not inferior to that of frozen section diagnosis by wedge resection in the FSA group. In any case, it is important for surgeons to recognize the possibility of diagnostic error, even with FSA by wedge resection. FSA should be considered for cases likely to be benign to avoid unnecessary lobectomy. Therefore, DL is an acceptable treatment option for large or deep nodules and GGNs selected carefully with malignant clinical diagnosis when performing a preoperative nonsurgical biopsy can expose the patient to unnecessary risks or inadequately lengthen surgical management.

## Disclosure

The authors have declared that no conflicts of interest exist.
